# The Effect of Cleft Lip on Adults' Responses to Faces: Cross-Species Findings

**DOI:** 10.1371/journal.pone.0025897

**Published:** 2011-10-10

**Authors:** Christine E. Parsons, Katherine S. Young, Emma Parsons, Annika Dean, Lynne Murray, Tim Goodacre, Louise Dalton, Alan Stein, Morten L. Kringelbach

**Affiliations:** 1 Department of Psychiatry, University of Oxford, Oxford, United Kingdom; 2 Centre of Functionally Integrative Neuroscience, Aarhus University, Aarhus, Denmark; 3 Department of Psychology, University of Bath, Bath, United Kingdom; 4 School of Psychology and Clinical Language Sciences, University of Reading, Reading, United Kingdom; 5 Spires Cleft Centre, John Radcliffe Hospital, Oxford, United Kingdom; University of Sydney, Australia

## Abstract

Cleft lip and palate is the most common of the congenital conditions affecting the face and cranial bones and is associated with a raised risk of difficulties in infant-caregiver interaction; the reasons for such difficulties are not fully understood. Here, we report two experiments designed to explore how adults respond to infant faces with and without cleft lip, using behavioural measures of attractiveness appraisal (‘liking’) and willingness to work to view or remove the images (‘wanting’). We found that infants with cleft lip were rated as less attractive and were viewed for shorter durations than healthy infants, an effect that was particularly apparent where the cleft lip was severe. Women rated the infant faces as more attractive than men did, but there were no differences in men and women's viewing times of these faces. In a second experiment, we found that the presence of a cleft lip in domestic animals affected adults' ‘liking’ and ‘wanting’ responses in a comparable way to that seen for human infants. Adults' responses were also remarkably similar for images of infants and animals with cleft lip, although no gender difference in attractiveness ratings or viewing times emerged for animals. We suggest that the presence of a cleft lip can substantially change the way in which adults respond to human and animal faces. Furthermore, women may respond in different ways to men when asked to appraise infant attractiveness, despite the fact that men and women ‘want’ to view images of infants for similar durations.

## Introduction

Cleft lip and palate is the most common of the congenital conditions affecting the face and cranial bones, with an incidence of 1 in 700 live births in the UK [1]. Previous studies have reported that infants with cleft lip and palate and their mothers are less responsive to each other than when the infant has no facial anomalies (e.g., [2], [3], [4], [5]). Critically, cleft lip and palate in infancy has been associated with a range of adverse outcomes in childhood, including behavioural, emotional and cognitive difficulties. Research suggests adverse outcomes in terms of child development, and especially cognitive outcomes, may be a consequence of early difficulties in mother-child interactions, and specifically a lack of maternal responsiveness that occurs where the infant has a cleft lip [4], [5]. These parent-infant difficulties are particularly likely in cases where the infant's face shows a high degree of disfigurement [5]. It is of considerable importance for clinical practice and intervention to establish why such difficulties in parent-infant interactions emerge. In older children and adults with cleft lip and palate, self perception of physical attractiveness has been shown to be related to a number of outcomes, such as self-esteem and psychological adjustment [6], [7], [8].

At the most fundamental level, interactions are built up from the parent and the infant recognising and responding to each other. Infant facial cues are central in this regard, and adults are remarkably attuned to the facial features that characterise their young (e.g., [9], [10]). We recently reported a pattern of early brain activity seen in response to unfamiliar infant faces but not for adult faces, perhaps reflecting a biological basis for this attunement to infants [11]. Women have long been credited with having a greater affinity for infants than men and greater skill in interacting with them, (e.g., [12]), but gender differences in responding to infants are far from clear cut (see [13] for a review). Women have been shown to be better at picking the ‘cuter’ of two infant faces morphed in their facial attractiveness [10], and tend to give infants higher attractiveness ratings than men [14]. For healthy infant images, men and women have been shown to ‘work’ at a similar rate (as indexed by key pressing) to view images [14], [15], but for infants with a range of facial abnormalities, there is some evidence to suggest that women will ‘work’ to remove the images more so than men [15]. Overall, findings from these studies have not been conclusive, but suggest that both men and women are sensitive to the physical features of infant faces. There is also evidence that infants with cleft lip are rated as less attractive than healthy infants [16], although this study did not examine the issue of potential gender differences.

We decided to explore how adults respond to infant faces with cleft lip and healthy infant faces for two main reasons. First, we considered that such a comparison would provide us with a window into understanding how adults respond to a disturbance to one region within the infant face. In contrast to other abnormalities that affect the face in a more global way (e.g., Foetal Alcohol Syndrome, Down's Syndrome, Williams Syndrome) infants with cleft lip have specific morphological abnormalities. The lip area is just one of a cluster of features that comprise what is colloquially termed ‘cuteness’. Notwithstanding differences in severity, the change to the facial configuration that occurs with a cleft lip is relatively constant across infants, affecting specifically the orofacial, and in some cases the nasal, areas. Second, if the presence of a cleft lip does disrupt the typical response of an adult to an infant face, it may help account for some of the difficulties in face-to-face interactions between infants with cleft lip and their parents. We asked whether adults would respond differently to unfamiliar infants with cleft lip compared with healthy infants and whether degree of cleft severity would modulate responses, as reported previously [5]. We also examined how adults respond to animal faces with cleft lip, as a method of investigating issues around the social acceptability of looking at human faces with an abnormality.

The predominant behavioural paradigm in the investigation of the attractiveness of facial features has required participants to consciously rate the attractiveness of infant faces. Such a paradigm does not tap into current understanding of the subcomponents underlying the evaluation of hedonic stimuli, which has been demonstrated to consist of at least three components, including hedonic appraisal (‘liking’), incentive salience (‘wanting’) and learning [17]. We therefore asked whether, beyond simple appraisal, viewing images of infant faces with and without cleft lip could differentially shape immediate behaviour in an experimental paradigm. In addition to a ‘liking’ task measuring the conscious appraisal, we used a key press ‘wanting’ task to examine the amount of work participants would perform in order to change the relative duration they viewed an individual image for (see [18], [19], [20]), given recent findings of gender differences across these two measures [14].

## Experiment 1

### Ethics statement

This study was approved by the Oxfordshire Research Ethics Committee (12/07/2010). Participation was voluntary and all participants gave written informed consent.

### Participants

Twenty men and 20 women participated in Experiment 1. Participants were recruited from the student and general population through poster advertisement. Inclusion criteria for participation were: normal vision, or vision corrected to normal, no medication affecting the brain and no experience of caring for an infant with cleft lip. Five of the men and three of the women were parents. The age range of the participants was between 18 and 35 years (M = 24, SD = 6).

### Stimuli

**Figure 1:**
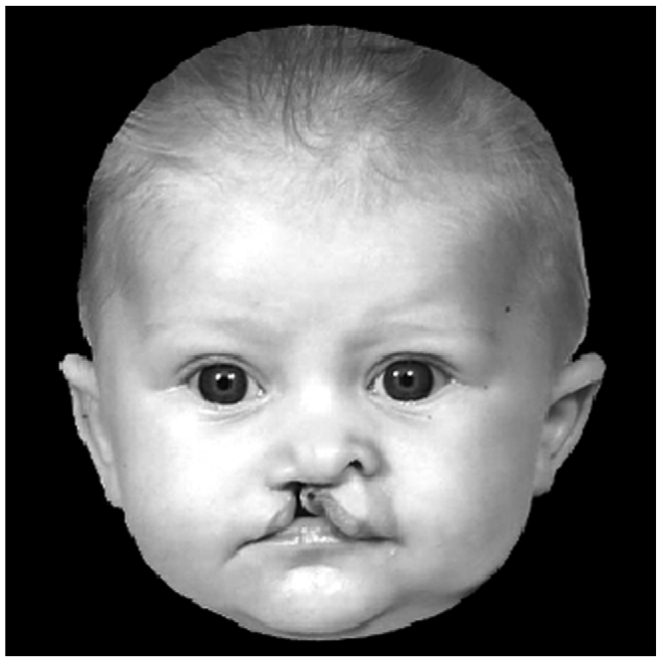
Example of an image of an infant with cleft lip.

Stimuli consisted of a total of 63 images of infants; 38 were healthy infants and 25 infants had cleft lip. The healthy infant images were obtained from a standardised database described elsewhere [11]. Additional face stimuli were obtained from a number of parents of young infants, and were matched to the original healthy infant faces. Parental permission was obtained for the use of all infant images in this study. We compiled a comparable set of images of infants with cleft lip (see Figure 1), again with parental permission for the use of the images. Both sets of infant faces were selected such that each infant was facing forward with eyes fully opened, a comparable direction of eye gaze and a neutral emotional expression. To represent all types of cleft, we chose images of infants with both unilateral and bilateral clefts, and within these two categories, complete and incomplete clefts. The use of all images in this study was approved by the Oxford Research Ethics Committee.

In order to select appropriate stimuli for this task, a panel of 56 adults (30 females, 26 males) rated the emotional valence of each infant face with cleft lip and also the severity of the cleft. Two scales were used: one to obtain ratings of emotional valence (1 = happy, 2 = neutral, 3 = sad), and the other for cleft severity (1 = mild, 2 = moderate, 3 = severe). Participants were asked to rate the valence within each infant face, rather than the valence induced by looking at each infant face. All faces were rated by the majority of participants as neutral (M = 2.03, SD = 0.44), but there was a wide range of cleft severity ratings. Six of the infants faces were rated as having a mild cleft lip (1.00–1.51), nine of the faces were rated as having a moderate cleft lip (1.62–2.49) and 11 were rated as having a severe cleft lip (2.55–2.93). The images were digitized at 600 dpi in 8-bit greyscale, and cropped to 300 pixels wide to 300 pixels high using Gimp 2.6.8 software (GNU Image Manipulation Program, 2008). All images were presented in greyscale and were matched for size and luminosity. Participants viewed the faces on a computer monitor, such that face stimuli subtended a visual angle of approximately 4x2 degrees.

### Methods

We used two measures, a ‘liking’ and a ‘wanting’ task, to capture the dual aspects of appraisal and incentive salience in adults' hedonic processing of the infant faces, described in Parsons et al. [14]. The appraisal task required participants to rate the attractiveness of the faces, providing a measure of ‘subjective liking’ of the images, similar to the task we have used extensively for measuring ‘liking’ of other hedonic stimuli (e.g., [21]). The ‘wanting’ or ‘key press’ task required participants to key press to either increase or decrease the relative viewing duration of each image. This task probed the incentive salience or ‘wanting’ to view the faces by measuring the amount of work participants are willing to do (and the resultant viewing times) in response to each stimulus, which in some respects was similar to other keypressing tasks [15], [19], [20], [22].

In both tasks the participants were presented with a face image on the centre of the screen and a vertical visual analogue scale (VAS) immediately to the right. In the ‘liking’ task, the VAS ranged from +4 ‘Very attractive’ to -4 ‘Very unattractive’ and the participants were asked to rate the attractiveness of images of the infant faces. Each stimulus was presented for five seconds and participants rated the 63 stimuli twice each. The order of stimuli was pseudorandomised across participants, by creating four versions of the task with different stimuli orders in each version. Ten participants completed each version. The order of completion of the ‘wanting’ and ‘liking’ task was also counterbalanced across participants.

In the ‘wanting’ task, the default viewing time of each stimulus was 6 seconds and participants could adjust this viewing time according to their ‘work-effort’, i.e. the frequency of key-pressing of either the ‘up’ or the ‘down’ keys. The visual analogue scale, again presented on the right of each stimulus, provided participants with a real time indication of the viewing time duration similar to an egg timer, with a bar moving downwards over time (the speed of movement could either be slowed or increased by the key presses). Participants were also told that the key press task would last for a set duration overall, independent of the viewing time of each individual image, such that if they chose to remove images from the screen, they would see a greater number of images in total, or if they chose to view images for longer, they would see fewer images overall in total In both tasks, participants responded using the index finger of their dominant hand.

### Results

**Figure 2:**
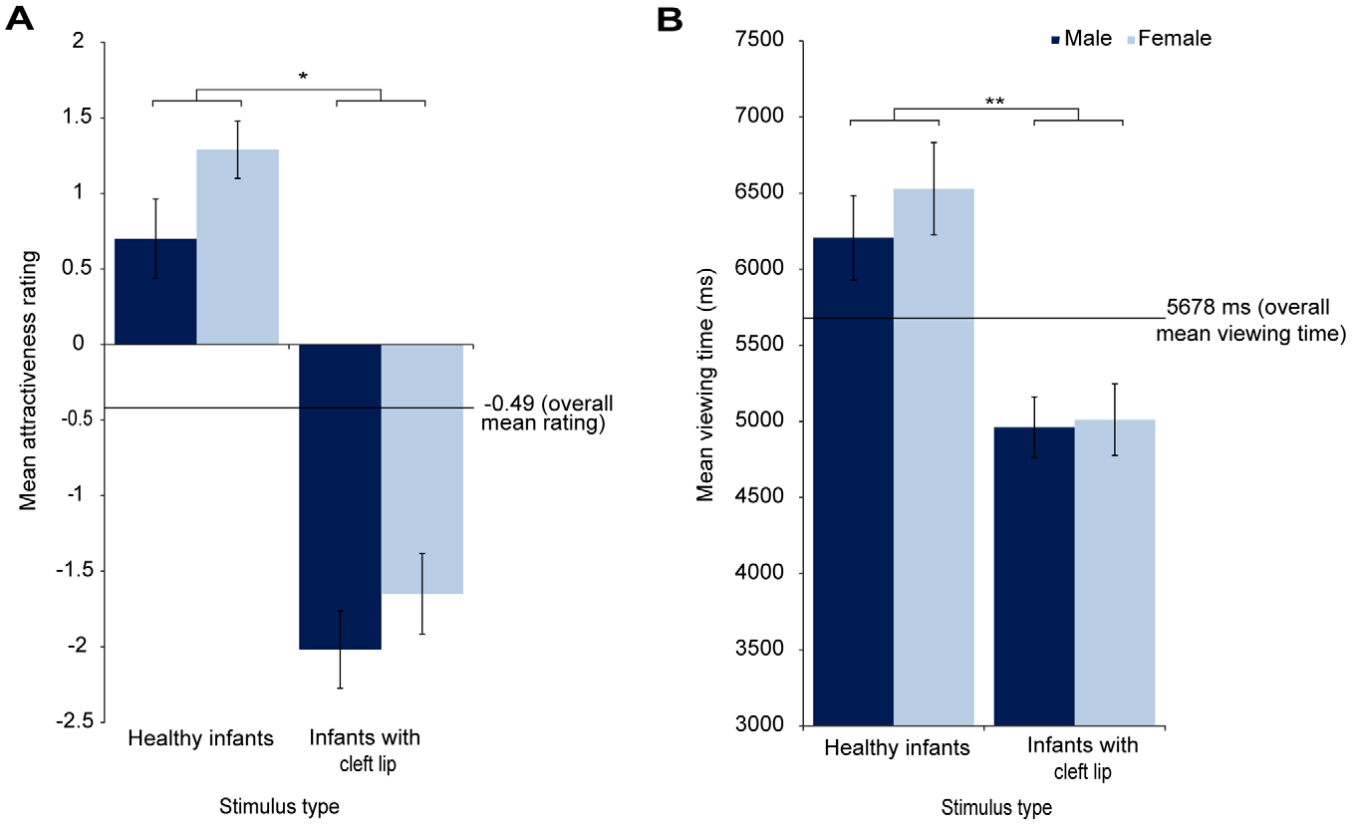
‘Liking’ and ‘wanting’ responses to healthy infants and infants with cleft lip. Left, images of healthy infants were rated as significantly more attractive than images of infants with cleft lip. Overall attractiveness ratings (of healthy infants and infants with cleft lip) were significantly higher for women than men. Right, images of healthy infants were viewed for significantly longer than images of infants with cleft lip. There were no significant gender differences in viewing times. Error bars represent the mean +/− standard error, * p<0.001, ** p<0.0001.

Analyses were conducted using the viewing times and attractiveness ratings averaged across exposures in SPSS (17.0). Viewing times were measured to millisecond accuracy, and attractiveness ratings (measured on a VAS ranging from +4 to −4) were recorded with equivalent precision, to 2 decimal places. Figure 2 presents the viewing times and attractiveness ratings for the infants with and without cleft lip by participant gender. A 2 X 2 mixed ANOVA was used to analyse the attractiveness ratings, with image type (infant with cleft, healthy infant) as the within-participants factor and gender (male, female) as the between-participants factor. Two clear patterns emerged from our analyses of the attractiveness ratings. First, participants rated the images of infants with cleft lip as less attractive than infants without (F (1, 38) = 121.70, p<0.001). Second, compared to men, women rated infants as more attractive across the board (F (1, 38) = 4.19, p<0.05). There was no interaction between participant gender and infant category (F(1, 38) = 0.18, p = 0.67).

Consistent with the attractiveness ratings, infants with cleft lip had significantly shorter viewing times than infants without (F(1, 38) = 32.18, p<0.0001). However, in contrast to the attractiveness ratings, there were no differences between the men and women's viewing times (F(1, 38) = 0.46, p = 0.49). There was again no interaction between gender and infant category (F(1, 38) = 0.31, p = 0.58).

**Figure 3:**
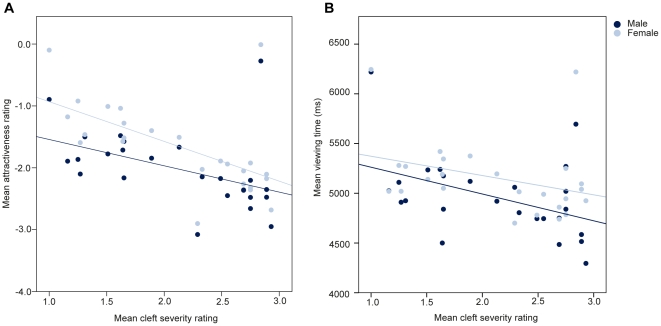
There was a significant correlation between previous ratings of cleft severity (1 = mild, 2 = moderate, 3 = severe) and participants' attractiveness ratings and viewing times of each infant image.

We also explored the relationship between prior subjective ratings of cleft severity (by an independent panel, see Methods) and our participants' responses to the infant images (see Figure 3). There was a significant relationship between the cleft severity ratings and participants' mean attractiveness ratings (r_s_  = −0.24, p<0.0001) and participants' mean viewing times (r_s_  = −0.12, p<0.0001). In order to examine whether these relationships differed across men and women, z-scores of the differences between the correlations for men and women were calculated for the attractiveness ratings and the viewing times separately. There were no significant differences in the correlation between cleft severity ratings and mean attractiveness ratings between men (r = 0.46) and women (r = 0.59; p = 0.49) or in the correlation of cleft severity with mean viewing times between men (r = 0.43) and women (r = 0.33; p = 0.7). Those infants with cleft lip that were rated as most severe (by an independent panel) were viewed for the shortest durations and received the lowest attractiveness ratings.

## Experiment 2

One evolutionary question that arises from the findings of Experiment 1 concerns the specificity of responses to human faces compared to faces from other species. Cleft lip is one of the rare facial abnormalities that affect animal and human facial structure in an analogous way. We chose to investigate whether the same abnormality, cleft lip, would impact upon the way adults respond to animal images, in the way it did for infant images. A possible explanation for the finding of shorter viewing durations for the infants with cleft lip in Experiment 1 is that it is considered socially unacceptable to look at a facial abnormality for an extended period of time. The use of animal faces with cleft lip allows us to address this possibility.

### Stimuli

**Figure 4:**
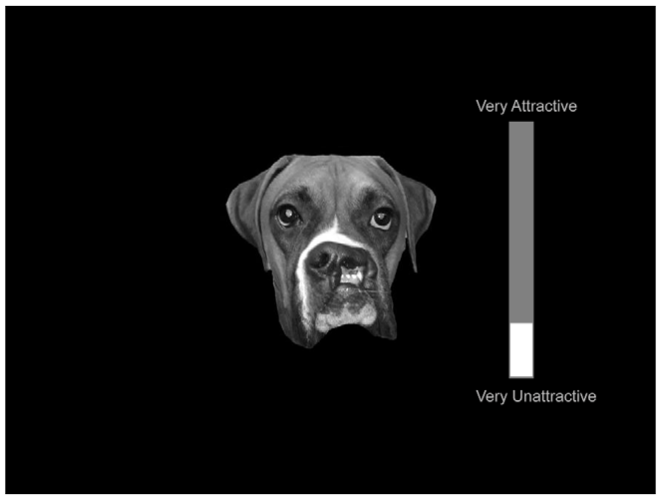
Example of an animal face with cleft lip, post-rating on the ‘liking’ task.

We acquired a set of animal faces with cleft lip from a number of veterinary surgeons and pet owners (see Figure 4). The rarity and poor survival rate of animals with the condition meant that only a limited number of images could be sourced. A total of 25 images of animals with cleft lip were used: five puppies, 14 dogs, one kitten and six cats. We also used 25 images of healthy animals; we included the same number of cats and dogs in the healthy animal category as in the animal with cleft lip category. In order to directly compare adults' responses to humans and animals with cleft lip, we included the infants with cleft lip in this task also. All face images have been converted to greyscale and matched for size and luminosity, such that they were comparable to the other face images. All other methods were identical to Experiment 1.

### Participants

Participants were healthy men (n = 20, two parents) and women (n = 23, four parents), with a mean age of 29 years (SD = 9) and an age range of between 20 and 60 years. Consistent with Experiment 1, participants were recruited from the student and general population through poster advertisement, and the same inclusion and exclusion criteria were used.

### Results

**Figure 5:**
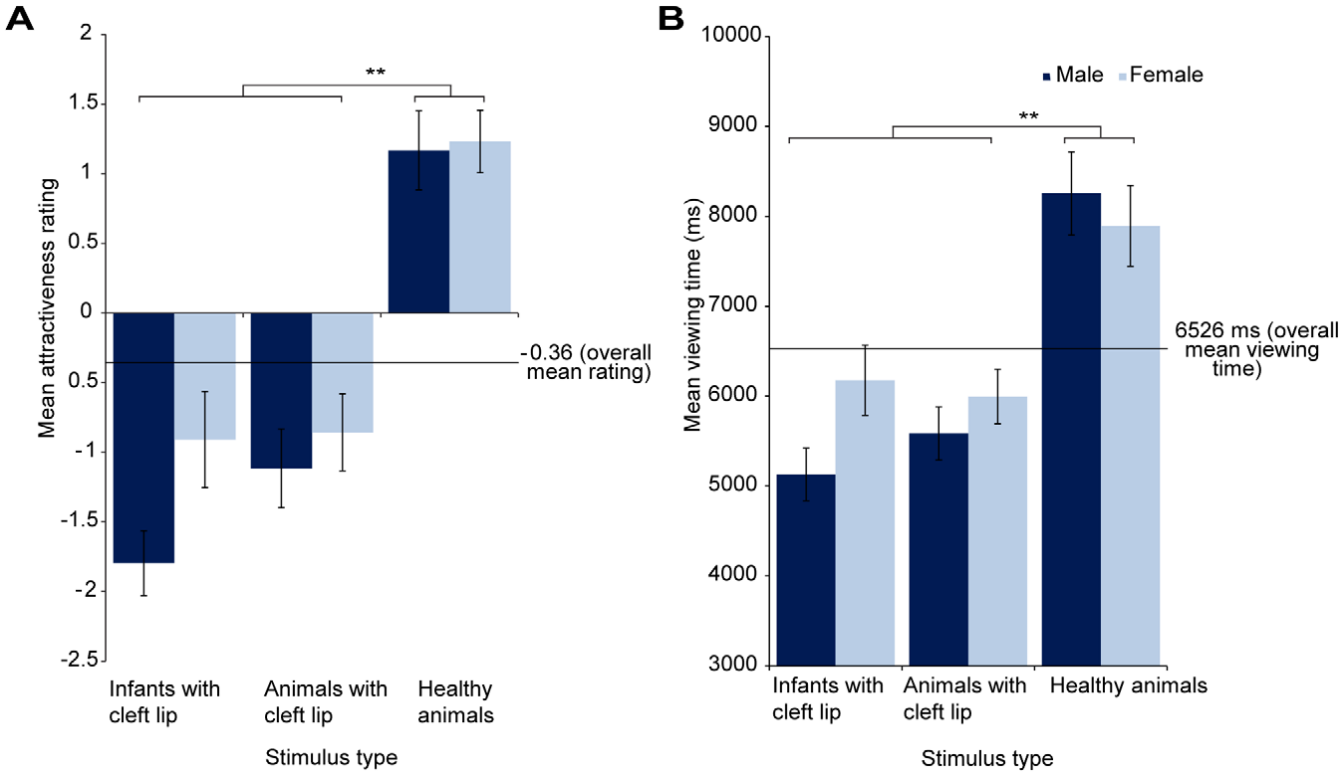
‘Liking’ and ‘wanting’ responses to infants with cleft lip, animals with cleft lip and healthy animals. Left, images of healthy animals were rated as significantly more attractive than images of animals with cleft lip and infants with cleft lip. Right, images of healthy animals were also viewed for significantly longer than images of animals and infants with cleft lip. Responses to infants with cleft lip and animals with cleft lip were remarkably similar across both measures. There were no significant gender differences in responses in this study. Error bars represent the mean +/− standard error, ** p<0.0001.

Figure 5 presents the viewing times and attractiveness ratings for the infants with cleft lip, the animals with cleft lip and the healthy animals. For the attractiveness ratings, there was a significant main effect of image type (F(1.6, 67) = 82.69, p<0.0001). Healthy animals received significantly higher attractiveness ratings than the animals with cleft lip (t(42) = 12.07, p<0.0001) and the infants with cleft lip (t(42) = 9.78, p<0.0001). The animals with cleft lip were rated as slightly more attractive than infants with cleft lip, but this difference only approached significance (t(42) = 1.99, p = 0.053). The main effect of gender (F(1, 41) = 0.57, p = 0.45) and the interaction effect of image type and gender (F(1.6, 67) = 1.12, p = 0.32) were not significant.

For the viewing time data, there was a significant main effect of image type (F(1.6, 64) = 39, p<0.0001). Healthy animals were viewed for significantly longer than the animals with cleft lip (t(42) = 7.21, p<0.0001), and the infants with cleft lip (t(42) = 6.35, p<0.0001), consistent with attractiveness rating data. There was no difference between the mean viewing time of the infants with cleft lip and the animals with cleft lip (t(42) = 0.69, p = 0.49). The main effect of gender (F(1, 43) = 0.03, p = 0.85) and the interaction effect (F(1.6, 64) = 1.95, p = 0.16) were not significant.

## Discussion

Our current findings demonstrate that disruption to just one facial feature can substantially change adults' responses to infant faces, underlining how sensitive adults are to the normal facial configuration. Adults viewed both human infants with cleft lip and animals with cleft lip for shorter durations than the healthy infants and healthy animals. Infants and animals with cleft lip were also rated as less attractive than the healthy infants and animals. The human infants with more severe cleft lips (as rated by an independent panel) were viewed for shorter durations and rated as less attractive than those infants with less severe cleft lips.

In Experiment 1, we found an interesting difference between men and women in their ratings of infant attractiveness. All stimuli presented were images of infants; women gave higher attractiveness ratings than men overall, but no differences emerged between men and women's ratings of infants with cleft lip or healthy infants when considered separately. In Experiment 2, where stimuli included images of infants with cleft lip, animals with cleft lip, and healthy animals, there were no significant differences between men and women's attractiveness ratings, indicating that women were not simply rating all faces as more attractive than men. In Experiment 1, while we did find a significant difference between men and women's attractiveness ratings of the infant faces, we failed to find any differences in men and women's viewing durations of these faces, or indeed any of the categories of faces. This suggests that women's explicit appraisal of infant attractiveness (‘liking’) was more positive than men's, but their willingness to work to view the images (‘wanting’) was similar. This is consistent with our previous findings demonstrating that women rate healthy infants as more attractive than men, despite similar elected viewing durations [14].

Women's higher ratings of infant attractiveness is broadly consistent with work demonstrating that women are more sensitive to infant facial ‘cuteness’ (e.g., [10]). However, compared to men, we did not find that women rated the infants with cleft lip as more attractive or viewed any images of infants for longer. One previous study of men and women's responses to faces with a range of abnormalities found that women exerted more effort to remove these images than men [15], a finding that is at odds with our results. If anything, the women tested here had slightly (although not significantly) longer viewing times of the infants with cleft lip. The reason for this discrepancy in findings is unclear, but may be related to the fact that we examined women's responses to one specific facial abnormality, while the Yamamoto et al. [15] study examined responses to a range of abnormalities, from superficial skin disorders to global structural changes such as that seen in foetal alcohol syndrome. It should be noted that the sample size tested here was more than twice as large as that included in by Yamamoto et al.

No differences emerged between adults' responses to the human infants with cleft lip and the animals with cleft lip on either the attractiveness ratings or the viewing time measure. The presence of a cleft lip was associated with more negative appraisal of attractiveness and shorter viewing durations whether it occurred in a human or domestic animal. It may be that images of domestic animals, for whom adults regularly provide care, elicit ‘liking’ and ‘wanting’ responses that are not markedly different from those seen for human infants, at least as measured here. This provides us with some insight into the nature of our frequent attachment to domestic animals.

Our findings indicate that the presence of a cleft lip alters the typical response of an adult to an unfamiliar infant face or animal face. Our participants chose to view the images of infants and animals with cleft lip for shorter durations and rated the faces as less attractive relative to the healthy comparison faces, an effect that was especially strong in the infants with very severe cleft lip. We tested a population with no experience of caring for an infant with a cleft lip in order to investigate general responsivity, and not responsivity to one's own infant. This is, in a sense, a limitation of this work: it remains to be seen how these experimental measures of attractiveness appraisal and motivational salience translate into actual interactions with an infant with cleft lip. There is some evidence to suggest that parental status can impact upon adults' physiological responses to infants (e.g., [23], [24]). In light of such findings, it would be of interest to examine whether parenting experience alters responses to infant faces as measured here. Nonetheless, these findings are consistent with studies demonstrating difficulties in interactions between mothers and infants with cleft lip, particularly where the cleft lip is severe [4], [5].
